# The Brazilian Preference: Cesarean Delivery among Immigrants in Portugal

**DOI:** 10.1371/journal.pone.0060168

**Published:** 2013-03-26

**Authors:** Cristina Teixeira, Sofia Correia, César G. Victora, Henrique Barros

**Affiliations:** 1 Department of Clinical Epidemiology, Predictive Medicine and Public Health, University of Porto Medical School, Porto, Portugal; 2 Institute of Public Health – University of Porto, Porto, Portugal; 3 Federal University of Pelotas, Pelotas, Brazil; London School of Economics, United Kingdom

## Abstract

**Objective:**

To evaluate how the country of origin affects the probability of being delivered by cesarean section when giving birth at public Portuguese hospitals.

**Study Design:**

Women delivered of a singleton birth (n = 8228), recruited from five public level III maternities (April 2005–August 2006) during the procedure of assembling a birth cohort, were classified according to the country of origin and her migration status as Portuguese (n = 7908), non-Portuguese European (n = 84), African (n = 77) and Brazilian (n = 159). A Poisson model was used to evaluate the association between country of birth and cesarean section that was measured by adjusted prevalence ratio (PR) and respective 95% confidence intervals (95%CI).

**Results:**

The cesarean section rate varied from 32.1% in non-Portuguese European to 48.4% in Brazilian women (p = 0.008). After adjustment for potential confounders and compared to Portuguese women as a reference, Brazilian women presented significantly higher prevalence of cesarean section (PR = 1.26; 95%CI: 1.08–1.47). The effect was more evident among multiparous women (PR = 1.39; 95%CI: 1.12–1.73) and it was observed when cesarean section was performed either before labor (PR = 1.43; 95%CI: 0.99–2.06) or during labor (PR = 1.30; 95%CI: 1.07–1.58).

**Conclusions:**

The rate of cesarean section was significantly higher among Brazilian women and it was independent of the presence of any known risk factors or usual clinical indications, suggesting that cultural background influences the mode of delivery overcoming the expected standard of care and outcomes in public health services.

## Introduction

European high-income countries are increasingly multi-ethnic societies where a large percentage of childbearing women were born abroad.[1]–[6] Their outcomes became key priorities for many governments as disparities in perinatal outcomes between foreign-born and native population have been reported, [7]–[12] suggesting inequities in access to and quality of health care. The magnitude and the pattern of such disparities differ according to the immigrants country of origin [7]–[10]_ENREF_2_ENREF_2_ENREF_2 and has been partially explained by barriers such as language [7], [11], [12] or the lower socioeconomic status of immigrants.[8], [11]

Cesarean section is one of the most debated pregnancy outcomes, being frequently used to evaluate the quality of obstetric care. The dramatic increase in cesarean rate over the last decades [13] has been a matter of public health concern due the increased risk of severe morbidity for mother [14]–[17] and child [17]–[19] associated with surgical delivery, in addition to the increased costs demanded by this mode of delivery.[20] In order to revert the upward trends in surgical delivery, it seems important to identify what groups of women undergo cesarean section and to investigate the underlying reasons.

The cesarean rate shows a distinct socioeconomic gradient with higher rates observed in private hospitals,[5], [21], [22] which suggests the influence of determinants other than clinical conditions. There is also a well documented wide international variation in cesarean rates that range from less than 1% in some African countries to more than 40% in Brazil, Dominican Republic or Cyprus.[23]_ENREF_10_ENREF_18_ENREF_17 While the main reasons for this disparity remain unclear, the study of women born in different countries but giving birth in the same host country is a particularly interesting situation to address this issue.

Research in European countries has emphasized differences in the mode of delivery not only between foreign-born and native women, but also according to the immigrant country of origin [2]–[6], [24]–[27] which could not be fully explained by socioeconomic status, or pregnancy complications.[2] The occurrence of such differences highlights the interplay of two important aspects. On one hand, cultural context shapes women's view and preferences regarding childbirth.[28] On the other hand, the cultural gap and linguistic barriers between the caregiver and the immigrant woman could lead to inaccurate obstetric evaluation and differential perception of obstetric risk by health care providers.[2], [24], [29]_ENREF_110 The interaction between both will dictate the decision about mode of delivery based on a set of non-medical factors, rather than by clinical indications. It is not clear whether the decision on mode of delivery is driven by patient's preferences or by cultural or language barriers between patients and their caregivers. Understanding these issues could provide insights into the decision-making process concerning mode of delivery in minority populations.

In Portugal, long term and settled migration has been linked to former colonial ties up to 1975, as consequence, in 2009 almost 50% of foreign-born residents in Portugal came from Brazil and Portuguese speaking African countries. [30] In this context, cultural gap or linguistic barriers are not expected for the majority of immigrant women once they shared with native women the same language and most often similar genetic and behavioral characteristics. We do not expect disparities in socioeconomic position between immigrant and native Portuguese women would create disparities in reproductive outcomes. Indeed, immigrants are entitled to use organized National Health System funded by public resources where the first contact is the GP/family doctor within the primary care centre, from which patients have access to higher levels of care if needed. In regard to reproductive health the Portuguese health care system provides prenatal, obstetric, neonatal and pediatric services free of charge for all childbearing women (citizen or foreign-born) and their children.[31]

Given the wide variability in cesarean section rates across geographical regions and the particular aspects concerning immigrant population in Portugal, the study of women from different countries giving birth in Portugal provides a particularly interesting opportunity to investigate how individual cultural heritage, local health care organization and medical decision may affect the mode of delivery. Thus, we aimed to assess the effect of the country of origin on the occurrence of cesarean delivery using a sample of pregnant women who were entitled to free care and delivered in five large public maternities in the North of Portugal.

## Methods

This study used baseline information obtained during the recruitment of a birth cohort assembled in the North of Portugal (Generation XXI). Women delivered in the five public maternities of the Porto Area, between April 2005 and August 2006, were invited to participate. All these hospitals offer the highest level of obstetric care and neonatal support, so they are classified as level III. In all, 70% of the eligible mothers were invited on the basis of “first come first served” and 8% of those invited refused to participate.The final sample comprised 8495 women. Information on social and demographic characteristics, obstetric and gynecological history, lifestyles and current pregnancy events was obtained using a structured questionnaire, through individual interview performed 24 to 72 hours after delivery by trained interviewers. Data on delivery and newborn characteristics were abstracted from medical records.

The study was approved by the Ethics Committee of the University of Porto Medical School/Hospital S. João and written informed consent was obtained from each participant.

A woman was classified as immigrant if (a) she was born abroad and both parents were foreign-born or (b) if one or both parents were Portuguese-born but she moved to Portugal at the age of 18 or later. Otherwise the participant was considered Portuguese. Immigrant women (n = 320) were classified into three groups according to the country of birth: European other than Portuguese (n = 84; 1.0%), African (n = 77; 0.9%) and Brazilian (n = 159; 2.0%). The mode of delivery was dichotomized as vaginal or caesarean section.

We excluded twin pregnancies (n = 144) and women with no information about migration status (n = 87). Also, to obtain groups as homogeneous as possible according to country of origin and large enough to allow statistical analysis, we excluded women born in Asia (n = 5), North-America (n = 1), and also African (n = 11) and South American (n = 19) women from countries where Portuguese is not the official language. Thus we considered for analysis 8228 mothers of singleton babies. These exclusion criteria also ensured that language barriers did not affect the choice of delivery methods.

We considered as potential confounders or modifiers of the association between country of birth and mode of delivery the maternal age (<25, 25–34 and > = 35 years), family monthly income ( = <1000€, 1001–1500€, >1500€), past obstetric history (primiparous, multiparous without previous caesarean section and multiparous with previous caesarean section), pre-pregnancy body mass index (<18.5, 18.5–24.9, 25.0–29.9 and > = 30 Kg/m^2^), chronic diseases previous to the current pregnancy (hypertension, diabetes and heart, respiratory and renal diseases), gestational age at first antenatal visit ( = <12 vs. >12 weeks), antenatal health care provider (private vs. public), pregnancy complications (gestational diabetes, pyelonephritis, hypertensive disorders and placental disorders), mechanism of labor onset (not induced vs. induced), fetal presentation (cephalic vs. non-cephalic) and birth sex-specific weight by gestational age (<10^th^ percentile, > = 90^th^ percentile, and otherwise)[32].

Missing data in each category of the covariates were not considered to compute the proportions but the percentage of missing values is provided.

Given the cross-sectional design of this study, the influence of country of birth on mode of delivery was evaluated using Poisson models to avoid overestimation of measures of association.[33] Data were presented as prevalence ratio (PR) and 95% confidence interval (95% CI). A baseline model was fitted containing country of origin and using Portuguese born women as reference. Maternal age was forced in the model. All other covariates were individually checked using manual forward addition and backward deletion and kept on final model if they changed the PRs of cesarean section by country of birth at least 10%. Interactions between country of birth and the other variables were also checked. Statistical analysis was performed with SPSS 19.0 software and the level of significance was set at p<0.05.

## Results

A cesarean section was performed on 2932 (35.6%) women, from which 891 (11% of all deliveries) were performed before labor onset. As shown in Table 1, the rate of this obstetric intervention varied significantly with country of birth, from 32.1% among European non Portuguese women to 48.4% among Brazilian (p = 0.007).

**Table 1:** Social demographic and obstetric characteristics by country of origin.

	Portuguese	European	African	Brazilian	p-value*
n (%)	7908 (96.1)	84 (1.0)	77 (0.9)	159 (2.0)	
	n (%)				
Hospital					
1	1868 (23.6)	20 (23.8)	22 (28.6)	33 (20.8)	0.362
2	1327 (16.8)	16 (19.0)	11 (14.3)	25 (15.7)	
3	843 (10.7)	7 (8.3)	10 (13.0)	12 (7.5)	
4	1935 (24.5)	22 (26.2)	11 (14.3)	52 (32.7)	
5	1935 (24.5)	19 (22.6)	23 (29.9)	37 (23.3)	
Maternal age (years)					
<25	1603 (20.3)	11 (13.1)	12 (15.6)	37 (23.3)	0.003
25–34	4847 (61.4)	65 (77.4)	43 (55.8)	104 (65.4)	
> = 35	1453 (18.5)	8 (9.5)	22 (28.6)	18 (11.3)	
% missing	0.1	0.0	0.0	0.0	
Family income (euros/month)					
= <1000	2769 (40.5)	17 (23.9)	34 (54.0)	71 (52.6)	<0.001
1001–1500	1981 (28.9)	23 (32.4)	10 (15.9)	26 (19.3)	
>1500	2094 (30.6)	31 (43.7)	19 (30.2)	38 (28.1)	
% no report/missing	13.4	15.5	18.2	15.1	
Parity and previous c-section					
Primiparous	4407 (55.7)	57 (67.9)	37 (48.0)	104 (65.4)	0.011
Multiparous no c-section	2488 (31.5)	21 (25.0)	29 (37.7)	32 (20.1)	
Multiparous c-section	1013 (12.8)	6 (7.1)	11 (14.3)	23 (14.5)	
Body Mass Index (Kg/m^2^)					
<25	4956 (68.7)	64 (83.1)	48 (72.7)	110 (77.5)	0.007
25.0–29.9	1591 (22.1)	10 (13.0)	14 (21.2)	29 (20.3)	
> = 30	663 (9.2)	3 (3.9)	4 (6.1)	3 (2.1)	
% missing	8.8	8.3	14.3	10.7	
Chronic pre-pregnancy disease					
yes	1000 (12.7)	8 (9.5)	10 (13.0)	14 (8.8)	0.416
no	6892 (87.3)	76 (90.5)	67 (87.0)	145 (91.2)	
% missing	0.2	0.0	0.0	0.0	
Gestational age at 1^st^ prenatal visit					
= <12 weeks	6620 (88.9)	69 (85.2)	57 (78.1)	129 (84.3)	0.007
>12 weeks	830 (11.1)	12 (14.8)	16 (21.9)	24 (15.7)	
% missing	5.8	3.6	5.2	3.8	
Private prenatal care					
yes	2886 (38.1)	38 (47.5)	17 (23.0)	36 (23.4)	<0.001
no	4693 (61.9)	42 (52.5)	57 (77.0)	118 (76.6)	
% missing	4.2	4.8	3.9	3.1	
Pregnancy complications					
yes	909 (11.5)	7 (8.4)	6 (7.8)	13 (8.2)	0.319
no	6962 (88.5)	76 (91.6)	71 (92.2)	146 (91.8)	
% missing	0.5	1.2	0.0	0.0	
Labour onset					
spontaneous	4937 (63.9)	55 (65.5)	43 (59.7)	101 (64.7)	0.757
induced	1930 (25.0)	23 (27.4)	20 (27.8)	34 (21.8)	
caesarean before labour	855 (11.1)	6 (7.1)	9 (12.5)	21 (13.5)	
% missing	2.4	0.0	6.5	1.9	
Fetal Presentation					
non-cephalic	449 (5.8)	1 (1.2)	1 (1.3)	8 (5.1)	0.116
cephalic	7339 (94.2)	81 (98.8)	74 (98.7)	149 (94.9)	
% missing	1.5	2.4	2.6	1.3	
Gestational age (weeks)					
<37	603 (7.6)	5 (6.0)	6 (7.8)	10 (6.3)	0.476
37–40	6769 (85.6)	69 (82.1)	68 (8.3)	134 (84.3)	
> = 41	528 (6.8)	9 (10.7)	3 (3.9)	15 (9.4)	
% missing	0.1	1.2	0.0	0.0	
Birthweight for gestational age					
Small (<10^th^ percentile)	1182 (15.0)	8 (9.6)	5 (6.5)	13 (8.2)	0.002
Large (> = 90^th^ percentile)	305 (3.9)	6 (7.2)	3 (3.9)	13 (8.2)	
Adequate (otherwise)	6393 (81.1)	69 (83.1)	69 (89.6)	133 (83.6)	
% missing	0.4	1.2	0.0	0.0	
Mode of Delivery					
vaginal	5108 (64.6)	57 (67.9)	49 (63.6)	82 (51.6)	0.007
cesarean section	2800 (35.4)	27 (32.1)	28 (36.4)	77 (48.4)	

* p-value for chi-square test; missing values excluded from the statistical analysis

Demographical, clinical and health care characteristics were also significantly different according to maternal country of origin. African women were older, less often primiparous and more frequently had their first antenatal visit after 12 weeks of gestation. Both Brazilian and African reported lower family incomes and used private antenatal care less frequently. Portuguese women were more frequently obese and presented higher proportion of chronic pre-pregnancy diseases. The higher proportion of babies large for gestational age was observed among non-Portuguese European and Brazilian women.

As shown in Table 2, compared with Portuguese women, Brazilian were significantly more likely to experience a cesarean delivery (PR = 1.26; 95%CI: 1.08–1.47) after adjustment for maternal age and past obstetrical history. No such effect was found for non-Portuguese European (PR = 0.91; 95%CI: 0.69–1.22) or African (PR = 1.02; 95%CI: 0.79–1.32) immigrants. The interaction between country of origin and parity was not statistically significant (p = 0.089), nevertheless we decided to stratify the results by parity. Accordingly, the adjusted prevalence ratio favoring cesarean section were higher in primiparous Brazilian women (PR = 1.19; 95%CI: 0.97–1.47) and particularly higher among multiparous both before (PR = 1.79; 95%CI: 1.40–2.29) and after adjusting for previous cesarean-section (PR = 1.39; 95%CI: 1.12–1.73). Also, Brazilian were more likely to have a cesarean section performed either before labor onset (PR = 1.43; 95%CI: 0.99–2.06), or during labor (OR = 1.30; 95%CI: 1.07–1.58).

**Table 2:** Risk of cesarean section and country of birth.

	All PR* (95% CI)	Parity		Timing of caesarean section	
		Primiparous PR† (95% CI)	Multiparous PR‡ (95% CI)	Before labour PR* (95% CI)	During labour PR* (95% CI)
Portuguese	reference	reference	reference	reference	reference
Non-Portuguese European	0.91 (0.69–1.22)	0.84 (0.58–1.22)	1.15 (0.79–1.66)	0.84 (0.41–1.75)	0.95 (0.67–1.33)
African	1.02 (0.79–1.32)	0.97 (0.66–1.44)	1.09 (0.79–1.51)	1.13 (0.68–1.89)	0.98 (0.68–1.41)
Brazilian	1.26 (1.08–1.47)	1.19 (0.97–1.47)	1.39 (1.12–1.73)	1.43 (0.99–2.06)	1.30 (1.07–1.58)

* Adjusted for maternal age, parity and previous c-section.

† Adjusted for maternal age

‡ Adjusted for maternal age and previous caesarean section

## Discussion

In the present study we compared the frequency of cesarean delivery in Portugal among women with different countries of birth. Brazilian immigrants presented the highest prevalence of cesarean delivery, either before or during labor, this was particularly evident among multiparous women. Our finding suggests that the cultural background might play an important role in the mode of delivery as this association was independent of the known clinical determinants of cesarean section and public hospitals follow common rules.

Research in European countries has emphasized differences in the mode of delivery between foreign-born and native women and also according to the immigrant country of origin.[2]–[6], [24]–[27] Higher rates of cesarean section (or both cesarean and operative vaginal delivery) have been reported among South-Americans,[2]–[6] more specifically Brazilian.[4], [6]_ENREF_12 Our results confirm these findings. However, except for one study,[5] linguistic barriers and a cultural gap between women and caregivers could be in place, explaining the disparities in obstetric outcomes. Such confounders were not expected for Brazilian women in Portugal because they share the same language and often very similar genetic and behavioral backgrounds.

A report of mode of delivery covering approximately 90% of births in developing world found 2.9% cesarean births in sub-Saharan Africa compared to a rate of 26% in Latin America and 27% in East Asia.[34] The high rates of cesarean section reported in South American countries are a matter of public health concern.[35] Between 2003 and 2004 the national cesarean rate among primiparous delivered of a singleton birth in Brazil reached 45.8%.[36] In 2007, cesarean sections constituted 47.0% of all deliveries in Brazil and almost half were scheduled in advance.[37] This extremely high prevalence seems to be a cultural consequence of attitudes towards labor and the perception of obstetric care among Brazilian women. The majority of Brazilian women perceive cesarean as the most adequate mode of delivery and as a symbol of high social status.[38], [39]_ENREF_29_ENREF_29 Cesarean rates among South American giving birth in European countries are higher than the ones observed among their native counterpart but its magnitude varies with the national rate among native women. Brazilian and other South American women who migrate to Norway presented 24% caesarean deliveries and this prevalence was twofold higher than the one observed for native Norwegian women (12%).[6] Another study reported 27.3% cesarean deliveries among Latin American women in Finland whereas 15.8% was the prevalence among native Finish women.[3] In Switzerland, 42% of deliveries in Brazilians were found to be surgical, while the proportion was 26% among Swiss born women.[4] In our study the prevalence of cesarean section was 35.4% among Portuguese and 48.4% among Brazilian women.

Overall these findings support the hypothesis that the mode of delivery is influenced by cultural aspects. The higher frequency of cesarean section among Brazilian and other South American whatever the host country considered, reflects the role of the cultural background that influences the knowledge and perception about consequences or risks of delivery,[39] corroborating the assumption that migrants bring their own perceptions and expectations about health care.[40] However, the fact that cesarean section rates in Brazilians and other South Americans are higher, but also vary according to the rate observed among native women in their respective countries, is likely the result of the interaction between the women's preferences regarding childbirth (rooted on their own cultural backgrounds) and the health care organization and obstetric care practices that they found in the host country, thus reflecting striking differences concerning the management of labor and delivery across European countries [41]

Within a context of medicalized childbirth, pregnant women and caregivers can negotiate the decision to perform a cesarean section.[29], [42], [43] Ambiguities in the evaluation of obstetric risks,[29] and the practice of defensive medicine[44] allow non-medical factors play a role in the decision-making process.[29], [42], [43] It has been reported that obstetricians in different European countries would perform a cesarean section in the absence of strict medical indication, simply because this is a woman's choice. Respect for the womańs autonomy and prevention of legal consequences linked to complications of vaginal delivery were the most frequently quoted reasons for such medical practice. [44] In the Portuguese National Health Service maternal request is not recognized as an acceptable indication for surgical delivery. Regardless of nationality, under similar conditions, within a context of free and universal health care as it happened in our study, pregnancy outcomes are expected to be the same. Our findings suggest that womeńs cultural beliefs about childbirth have probably driven in a subtle way the technical decision.

The American College of Obstetrics and Gynecology recommended that inter-hospital comparisons of cesarean rates should focus on nulliparous, term, singleton vertex women, as under optimal conditions the rates would be expected to vary minimally.[45] In our study, the higher risk of cesarean section observed in Brazilian was obvious in this situation, supporting the role of cultural negotiation, but it was more evident among multiparous. Lack of availability of birth analgesia, unpleasant experiences regarding delivery [39], [46]_ENREF_31 or concurrent tubal ligation[22], [47] explains higher demand for cesarean section among multiparous women delivering in Brazil. Unfortunately, we cannot judge whether these factors could differently influence cesarean section rate among women delivering in Portugal. Women from countries with high cesarean section rates are likely to have a previous cesarean section if previous deliveries occurred in the country of origin, enhancing the risk of this operation in the next deliveries. In our sample, almost half of multiparous Brazilian women had a previous cesarean section; this proportion is less than one-third among the other participants. However, the final prevalence ratios were adjusted for this and other previous experiences.

The prevalence of surgical delivery was similar in African and Portuguese born women. These findings are different from other studies that report higher prevalence of cesarean section or among African migrants than in native European women.[2]–[4], [6], [24]_ENREF_12 In those studies it was evident a large cultural gap between African women and receiving population; this factor increased the likelihood of differences in obstetric care as a consequence of less accurate caregiver's evaluation.[24], [29]_ENREF_19 In our study, African women came from former Portuguese colonial territories. This fact probably attenuated the expected differences as these immigrant women share most cultural characteristics with native Portuguese. The results from our study expectedly differed from those obtained in settings where cultural and linguistic differences between African and native women were obvious. Furthermore, 40% of these African women arrived in Portugal more than 10 years before their inclusion in this sample, which could also explain the absence of differences between African and Portuguese women (data not shown but available at request).

Studies addressing ethnic differences in mode of delivery provided no consistent results when foreign-born European women were compared with European native women. While Eastern European immigrants showed lower[3], [27]_ENREF_18 or similar[4]_ENREF_18 rates of surgical delivery compared with women from the host countries, Southern European women delivered in Switzerland had higher and Western Europeans had similar prevalence of caesarean delivery than native women.[4] In Sweden, Southern European women presented lower risk of non-normal childbirth but Western European immigrants were similar to Swedish women in terms of mode of delivery.[2] Some of these findings just probably reflect the small samples and too high heterogeneity to provide robust evidence. In our study, non-Portuguese European women constituted also a small and heterogeneous group, 25% came from Eastern, 17% from Southern and the remaining from Western _ENREF_13countries, but no differences regarding the mode of delivery were observed.

The main strength of this study is the large set of data on social and demographic characteristics, gynecological history and current pregnancy events obtained for these women, making available needed information about potential confounders for the association between womeńs country of origin and caesarean section. Also, our setting has unique characteristics, different from many European countries. Portugal only recently receives large migrant contingents but it is a country with a long tradition of emigration for Europe, Africa and America. Thus, our sample comprised descendent women from those emigrants. Because of that we decided to also classify as immigrant women born abroad with one or both Portuguese-born parents but only arriving in Portugal with 18 years or older. Doing so we have tried as much as possible to avoid misclassification not excluding women that were in fact exposed to a different cultural setting until to adulthood. The prevalence of cesarean section among this sub-sample was similar to other foreign natives and different from the Portuguese rate (data not shown).

The length of stay in Portugal for the immigrant women included in our sample was different according to the country of origin. Whereas 75% of Brazilian women arrived in Portugal less than five years before their inclusion in this sample, the length of stay for 80% of African women was more than five years (data not shown but available at request). In these circumstances it is difficult to disentangle whether the length of stay in the host country could influence our results.

In summary, we have found differences in the mode of delivery according to the country of birth that were not explained by demographic, medical or obstetrical risk factors, Brazilian women being significantly more likely to be delivered by cesarean section.

Portugal and other Western European countries report an increasing proportion of foreign-born women delivered in the host countries.[2]–[6] The deep understanding of the cultural determinants of the differences in caesarean uptake may improve perinatal care and the prevention and management of adverse perinatal events among immigrant women. Moreover, our results, allied with previous findings [5] highlight the importance of shaping the intervention strategies depending on the women's country of origin.
